# The short form endometriosis health profile (EHP-5): translation and validation study of the Iranian version

**DOI:** 10.1186/1757-2215-4-11

**Published:** 2011-07-27

**Authors:** Azita Goshtasebi, Maryam Nematollahzadeh, Fatemeh Zahra Hariri, Ali Montazeri

**Affiliations:** 1 Family Health Research Group, Mother and Child Health Research Centre, Iranian Institute for Health Sciences Research, ACECR, Tehran, Iran; 2 Mental Health Research Group, Mother and Child Health Research Centre, Iranian Institute for Health Sciences Research, ACECR, Tehran, Iran

## Abstract

**Background:**

Endometriosis Health Profile (EHP-5) is a valid instrument to measure health-related quality of life in endometriosis. This study was conducted to culturally adapt and validate the EHP-5 in Iran.

**Methods:**

Using a standard "forward-backward' translation procedure, the English language version of the questionnaire was translated into Persian (Iranian language). Then a sample of 199 women aged 18-50 years completed the questionnaire. To test reliability the internal consistency was assessed by Cronbach's alpha coefficient. Validity was evaluated using known groups comparison.

**Results:**

The mean age of respondents was 31.4 (SD = 5.4) years. Reliability analysis showed satisfactory result (Cronbach's alpha coefficient = 0.71). The questionnaire discriminated well between sub-groups of women differing in infertility and premenstrual syndrome (PMS) in the expected direction.

**Conclusion:**

This preliminary validation study of the Iranian version of the EHP-5 proved that it is an acceptable, reliable and valid measure of quality of life in endometriosis patients.

## Background

Endometriosis is defined as the presence of endometrial tissue (gland and struma) outside the uterus. The most frequent sites of implantation are the pelvic viscera and the peritoneum. Endometriosis is one of the most common chronic gynecological conditions that significantly affect 10-15% of women of reproductive age [1,2]. Typically a woman with endometriosis will experience various symptoms including chronic pelvic pain, dyspareunia and dysmenorrhea, dysuria, abnormal uterine bleeding or spotting and sub-fertility [3] and that endometriosis represents a serious risk factor for developing ovarian cancer [4].

Several studies have indicated that endometriosis could affect psychological and social well-being and thus lead to significant reduction in health-related quality of life (HRQoL) [5]. Disease-specific instruments are developed to assess the aspects of quality of life particularly affected by a disease or disorder [6]. Recently Jones et al. developed the Endometriosis Health Profile-30 (EHP-30) that is the first standardized, new disease-specific instrument evaluating the health-related quality of life in women with endometriosis [7]. The EHP-30 questionnaire contains a core questionnaire with 30 items and five scales: pain, feeling of control and powerlessness, emotional well-being, social support, and self-image. Six modular parts including 23 questions were also provided to measure the areas of sexual intercourse, work, and relationship with children, feelings about medical profession, treatment and infertility [8]. The authors of EHP-30 decided to produce a shorter form of the questionnaire. It might be less time consuming and more practical, easy to interpret information obtained by instrument, easier for respondents to complete short questionnaire than EHP-30. The Endometriosis Health Profile-5 (EHP-5) was developed as a short version of the original questionnaire [9]. The aim of this study was to develop and validate the Iranian version of EHP-5. There was no such an instrument available in Iran.

## Methods

### Translation and culture adaptation

Forward-backward procedure was applied to translate the English version of the EHP-5 into Persian (the Iranian language). Two independent professional translators produced two forward translations. Both translators were instructed to aim for conceptual rather than literal translation. Translators with one of the authors compared their translations and produced a single provisional version. Then two other professional translators translated the provisional questionnaire back into the English. The two translators were not aware of the questionnaire. Finally, an expert committee consisting of translators, the researchers, two midwifes, and one gynecologist and one epidemiologist reviewed all the translation and cultural adaptation processes were applied. They also evaluated the final English backward version with the original questionnaire. Consensus in terms of semantic, idiomatic, experiential, and conceptual equivalence was reached and a final version of the questionnaire (the Persian EHP-5) was provided. The final translated version of the questionnaire was given to 10 patients to complete and declare their understanding of the items to ensure face validity.

### Questionnaire

The EHP-5 contains 11 questions (items): five items including pain, control and powerlessness, emotional well-being, lack of social support, self image from the core questionnaire and six items from the modular questionnaire that may not be applicable to every woman with endometriosis including work, intercourse, and worries about infertility, treatment, and relationship with children and medical professionals. Each item is rated on a four-point scale (never = 0, rarely = 1, sometimes = 2, often = 3, always = 4 and not relevant if not applicable). Scores on the EHP-5 core and modular questionnaire then are transformed on a scale of 0 (indicating best possible health status) to 100 (indicating worst possible health status). If the 'not relevant' box was ticked for items on modular questionnaire the score could not be computed for that dimension.

### Sample and data collection

The final draft of the Iranian version of the EHP-5 was administrated to a sample of 199 women with a confirmed surgical diagnosis of endometriosis undergoing conservative surgery. All women were selected from two obstetrics and gynecology clinics in Tehran, Iran (Royan Institute and Avicenna Research Institute both affiliated to Iranian Academic Center for Education, Culture and Research).

The sample size calculation was based on an assumption that at least 10% of women in the reproductive age would suffer from endometriosis. Two trained female midwifes collected the data by face-to-face interview 1 to 12 months after diagnostic laparoscopy. All patients completed a questionnaire containing brief background information (such as age, marital and reproductive status, and family history) and the EHP-5 questionnaire. The study was carried out during July 2009 to March 2011.

### Statistical analysis

Internal consistency was assessed by calculating Cronbach's alpha coefficient. Value of 0.7 or greater was considered satisfactory [10]. Validity was assessed using known groups comparison to test how well the questionnaire discriminates between subgroups of the study sample that differed in reproductive health status. It was expected that women with infertility and PMS would have higher scores than women without infertility and PMS in all measures. Mann-Whitney U test was performed for comparisons. Women with infertility and PMS were identified after a confirmed diagnosis by gynecologists.

### Ethics

The study received ethical approval from the Iranian Institute for Health Sciences Research. The authors informed all women regarding the study objectives, and indicated that their participation is voluntary and they could withdraw at any time. Both oral and written instructions were given to patients to ensure that items were understood (i.e. there were no right or wrong answers to the questions and the participants should feel free and honestly state what they think), and the subjects were reassured about the confidentiality.

## Results

### The study sample

In all, 220 women were approached and 199 (90%) agreed to be interviewed. The main reason for those who did not participate in the study was dislike. The mean age of the respondents was 31.4 (SD = 5.4) years. Most were married (94.5%) and university educated (43.3%). The characteristics of the respondents are shown in Table 1.

**Table 1:** Demographic characteristics of the studied women (n = 199)

		No	%
**Age (year)**			

	18-25	26	13.1

	26-30	65	32.7

	31-35	65	32.7

	≥ 36 Mean (SD)	43 31.4 (5.4)	21.6

**Education**			

	Primary	15	8

	Junior high school	30	16

	High school	61	32.6

	University	81	43.3

**Marital status**			

	Single	9	4.5

	Married	188	94.5

	Widowed	2	1

**Employment status**			

	Employed	51	25.6

	Student	8	4

	Housewife	14	70.4

**Fertility status**			

	Fertile	35	17.6

	Infertile	164	82.4

**PMS**			

	Yes	60	30.2

	No	139	69.8

### Descriptive statistics and reliability

The descriptive statistics of the 5 items are shown in Table 2. The Cronbach's alpha coefficient was 0.71 for the instrument indicating a satisfactory result.

**Table 2:** Descriptive statistics for the EHP-5 core questionnaire

	Mean row scores (SD)	95% CI	Skewness	Response frequencies (%)				
				
				Never	Rarely	Some times	Often	Always
**Pain**	0.085 (0.07)	0.7-1	0.937	52.8	17.1	24.1	4	2

**Control & powerlessness**	1.44 (0.09)	1.26-1.62	-0.95	34.7	15.1	29.6	12.6	8

**Emotional well-being**	1.68 (1.3)	1.50-1.87	0.20	25.1	18.1	30.7	15.1	11.1

**Lack of social support**	1.37 (1.35)	1.18-1.57	0.511	38.7	16.6	22.1	13.6	9

**Self image**	1.48 (1.42)	1.27-1.68	0.375	40.2	12.6	17.1	19.1	11.1

### Known groups comparison

Known groups comparison was used to test the validity. It was hypothesized that women with infertility and PMS would have poorer quality of life than women without infertility and PMS. The analysis showed that the women with infertility had higher scores in pain, control and powerlessness, emotional well-being and self image and individuals suffering PMS had lower scores in pain, control and powerlessness, emotional well-being and lack of social support measures as expected (Table 3). This indicated that the EHP-5 well discriminated between subgroups of the people who differed in reproductive health status.

**Table 3:** known groups comparison for the EHP-5*

Infertility	No (n = 35)	Yes (n = 164)	
	**Mean (SEM)**	**Mean (SEM)**	P**

Pain	19.05 (2.01)	32.14 (4.29)	0.007

Control & powerlessness	34.14 (2.54)	45.0 (5.2)	0.07

Emotional well-being	38.87 (2.39)	57.85 (6.21)	0.002

Lack of social support	32.85 (5.95)	34.75 (2.62)	0.7

Self image	32.77 (2.74)	57.14 (6.02)	< 0.0001

**PMS**	No (n = 139)	Yes (n = 60)	

	**Mean (SEM)**	**Mean (SEM)**	P**

Pain	18.70 (2.16)	27.5 (3.44)	0.02

Control & powerlessness	31.47 (2.60)	46.66 (4.38)	0.002

Emotional well-being	37.76 (2.57)	52.5 (4.55)	0.003

Lack of social support	33.81 (2.88)	35.83 (4.34)	0.7

Self image	33.45 (2.97)	45.41 (4.91)	0.03

* The higher scores indicate worse conditions

** Derived from Mann-Whitney U test.

## Discussion

Although cross-cultural validation studies are very difficult to be carried out, their results might be considered worthwhile. Firstly, they provide standard health measures that make health status comparisons between different populations possible. Secondly, they provide validated instrument to monitor population health, estimate burden of disease and investigate outcomes in clinical practice and evaluate treatment effects. This was the first study on psychometric properties of the Iranian version of EHP-5 among an Iranian population. The results showed that the instrument was a reliable and valid measure that can be used in monitoring and measuring health-related quality of life of women with endometriosis.

Similarly the validity of the EHP-5 in different cultures was well documented. For instance, the finding from an English study showed that the instrument had good validity and could be applied among women with endometriosis [9]. Furthermore, a French version of the EHP-5 questionnaire has been developed and its acceptability and feasibility was desirable although validity was not reported [10].

Iranian version of the EHP-5 was extracted from its English version. The translation of the EHP-5 in Iran went through a rigorous method and was approved by the questionnaire's developers. Thus we did not encounter any difficulties in data collection.

The EHP-5 was basically designed to be a self-administrated questionnaire but it can be completed through an interview in person or by telephone [11]. However, face-to-face administration of questionnaire allowed the interviewers to collect data without any missing data. Although method of completing the EHP-5 has not been mentioned in its manual, the designers administered it by mail (self-administrated) and the rate of returning the questionnaire was reported to be 37.1% [9].

Reliability was assessed by internal consistency and validity was examined by known groups comparison. Cronbach's alpha coefficient showed a satisfactory result [12]. Known groups comparison indicated that the EHP-5 score were able to distinguish very well between subgroups of the respondents who differed in reproductive health status. The study showed that women with infertility and PMS had poorer health compared to women without infertility and PMS. These findings are consistent with results from other studies carried out in different countries [12-15]. However, there were no significant differences in social support between fertile and infertile or women with and without PMS. This might be explained by the fact that endometriosis by itself is a chronic disease and thus as it relates to social support, both fertile and infertile women or those with and without PMS showed a relatively similar scores and therefore one might not expect to find significant differences between women in this domain.

Tools assessing quality of life are being used in research and clinical trials rarely. For instance, only 17% of randomized trials assessed in a systematic review on the measurement of HRQoL in women had used standardized instruments [15]. In addition, often the instruments have been used in research just measured one dimension of illness e.g. psychological health status without identifying other areas of well being affected by disease [16]. Reasons for the limited use of health statues instruments in clinical setting are that they are too long and complicated for clinicians to understand and interpret the data gained by long health statues instruments, and also they are too burdensome for respondent to complete them [17,18]. The short form EHP-5 provides the chance of using a very brief instrument that measures health outcome for women with endometriosis where the long form version would not be appropriate. The results obtained by the EHP-5 from the analyses suggest this instrument provides the same picture of health-related quality of life as the longer version [9].

Although this study did not provide evidence for test-retest reliability, responsiveness to change or other tests; overall the findings showed that the Iranian version of EHP-5 is a reliable measure for measuring health quality of life in endometriosis patients. It will be especially useful in clinical settings where a short and economical endometriosis health status measure is needed. The future studies could focus on other psychometric properties of the EHP-5 questionnaire and also on different applications of the questionnaire as a recent study has suggested even it is a useful index in order to evaluate cost-effectiveness of healthcare interventions [19].

## Conclusion

This study presents the first step in evaluating psychometric properties of a well-known instrument measuring health-related quality of life of Iranian patients with endometriosis. Since health related quality of life was rarely assessed as primary end-point in studies of endometriosis in Iran, the Persian EHP-5 might possibly provide both clinicians and patients with numerous advantages as an important outcome measure in future studies. However, its sensitivity to change needs still to be studied.

## Competing interests

The authors declare that they have no competing interests.

## Authors' contributions

All authors were involved in designing of the study, data collection and analysis, interpretation of results and manuscript preparation. AG, MN and FZH prepared the first draft of the paper. AM and AG provided the final manuscript. All authors read and approved the final manuscript.
